# Differences in personality related determinants of empathetic sensibility in female and male students of medicine

**DOI:** 10.1371/journal.pone.0254458

**Published:** 2021-07-14

**Authors:** Barbara Bętkowska-Korpała, Roksana Epa, Karolina Sikora-Zych, Katarzyna Olszewska-Turek, Anna Pastuszak-Draxler, Anna Rajtar-Zembaty, Anna Starowicz-Filip

**Affiliations:** 1 Department of Medical Psychology, Jagiellonian University Medical College, Cracow, Poland; 2 Department of Clinical Psychology, University Hospital in Cracow, Cracow, Poland; Shahjalal University of Science and Technology, BANGLADESH

## Abstract

The issues of personality and its relations with the level of empathetic sensibility of medical doctors are broadly discussed in the literature. The aim of this study was an assessment of personality related predictors of empathy indicators in female and male students of medicine with consideration of gender differences. Methods applied were Empathic Sensitiveness Scale (ESS) and Personality Inventory (NEO-PI-R). The study included 153 participants, who were students of the fifth year of medical studies. Students filled in questionnaires during workshops in clinical psychological skills. Participation in the study was voluntary and anonymous. The statistical analysis was performed using Statistica 13 PL and PS IMAGO PRO (SPSS). Linear regression analysis with the interaction component was performed to explore the relationship between personality factors and gender and their interaction with the variable dependent level of empathy. The analysis showed that Extraversion, Openness and Agreeableness are associated with the level of Empathic Concern. Neuroticism, Extraversion, Agreeableness and Conscientiousness are associated with the level of Personal Distress. Extraversion, Openness, Agreeableness and Conscientiousness are associated with the level of Perspective-taking. The regression analysis with the interactive component showed that there is no relationship between gender and the level of empathy, therefore the interactions were insignificant. Empathetic sensibility is related to personality dimensions of the students of medicine. Although there has been no interaction among chief personality dimensions, empathy indicators and gender, detailed analysis of personality dimensions’ components has shown differences between men and women.

## Introduction

Enhancement of empathic attitude towards patients in medical doctors is one of the most important challenges in education [1, 2]. The American Association of Medical Universities in its guidelines stresses that empathy should be developed and evaluated in the course of medical studies [3, 4].

Davis defines empathy as a way of reacting to other people’s experiences and highlights its cognitive and emotional components. In his theory, a cognitive aspect of empathy (Perspective Taking, PT) refers to the ability of taking the perspective of another person. There are two affective components related to emotional reactions to suffering and painful experiences of other people. The first one, Empathetic Concern (EC) is related to the ability to experience interest and care, the second one, Personal Distress (PD) describes the degree to which an individual experiences discomfort and anxiety when faced with someone’s adverse experiences. Thus defined empathy and its dimensions can be measured by Davis’s questionnaire Interpersonal Reactivity Index (IRI) with its subscales: PT, EC, PD.

A number of correlations among various dimensions of empathy and personality traits have been observed [5–10]. A broadly recognised taxonomy of personality traits is the five-factor model (FFM). FFM defines personality as “part of a durable core of a person, a basis on which adjustment to continuous changes of life are made” [11]. FFM defines five chief personality factors, basic tendencies that are expressed via adjustment characteristics, such as empathic behaviours. Although these traits are biologically determined, they need to be considered as dispositions and not determinants. Each of the five dispositions–neuroticism, extraversion, openness, agreeableness and conscientiousness is described via its six characteristics. Basic tendencies are biologically determined but can also be influenced by an environment that changes biological mechanisms [12]. The more intense a factor is, the more likely the person will behave in a specific way. Interactions among factors, developmental and contextual factors will further influence the choice of certain behaviours [11]. FFM has a high explanatory potential of personality functioning, also due to the NEO-PI-R Personality Inventory that has been adopted in numerous countries.

Research on correlations between conceptualised, empathy and personality on the group of medicine students show that the results in PT (Perspective Taking) were positively correlated with z Agreeableness, Openness and Conscientiousness, and negatively correlated with Neuroticism and Extraversion. Higher results in EC (Empathetic Concern) were observed in subjects with higher Extraversion, Conscientiousness and Neuroticism. The higher the result in Neuroticism, the higher the level of PD (Personal Distress) in contact with suffering of others [6, 8].

Considering personality related factors influencing empathy, it needs to be taken into account that the level of empathy is heritable. Research shows that affective components of empathy are inherited to a higher extent (up to 52–57%) than the cognitive ones, which is related to biological components of personality [13].

In a number of research, the results show that women have a higher level of empathy than men in all its aspects, also in experiencing their own discomfort when faced with the suffering of others [13–15]. Other research carried out in a number of countries such as Portugal, South Korea, New Zealand, Iran and Poland do not confirm any differences in the level of empathy between men and women [1, 4, 16–20]. As the literature review gives mixed and ambiguous conclusions about the differences/ similarities in the level of empathy between men and women more research is needed on this issue.

### Aim

The aim of this study was an assessment of personality related predictors of empathy indicators in female and male students of the fifth year of medical studies.

## Materials and methods

### Ethics statement

All procedures followed were in accordance with the ethical standards of the responsible committee on human experimentation and with the Helsinki Declaration of 1975), as revised in 2000. Bioethics Committee of the Jagiellonian University has approved the study (nr of document: 1072.6120.175.2018). Informed and written consent was obtained from all individual participants included in the study.

### Study design and sample

Students of medicine have the opportunity to evaluate their style of psychological functioning during workshops in clinical psychological skills. They get to know, among others, indicators of their empathy and personality profile.

All the students of the fifth year of medicine participated in the study (n = 153; M = 57, F = 96; the mean age of study participants was 23 years). Participants who consented to participation in an anonymous study were filling in coded answer sheets.

### Measures

Two tools were applied in the research. Empathic Sensitiveness Scale (ESS) [7] and Personality Inventory (NEO-PI-R) [11, 12]. Additionally we inquired the gender. ESS have been created on the basis of Davis’s theory and the Interpersonal Reactivity Index (IRI) tool [21, 22]. ESS adaptation comprises three indicators: Empathetic Concern (EC), Personal Distress (PD) and Perspective Taking (PT). The authors of the Polish adaptation did not include the sub-scale of Fantasy. The EC indicator measures the tendency to express sympathetic affective reactions towards a person who experiences a difficult situation. The PD indicator shows the tendency to experience discomfort and unpleasant feelings in contact with a suffering person. Both above-mentioned indicators are affective. The third indicator—PT–a cognitive component of empathy measures the ability to spontaneously take the perspective of other people and understand their point of view. ESS has norms for sten scores for the Polish population [7].

Personality Inventory (NEO-PI-R) describes the adopted taxonomy of 5 chief personality factors: Neuroticism, Extraversion, Openness, Agreeableness and Conscientiousness. Each of the main factors consists of six subfactors. The inventory contains 240 items. Subjects use the Likert-type style (0 to 4) to indicate the truthfulness of a sentence in relation to themselves on an answer sheet. Raw results are calculated by means of keys and afterwards, taking into consideration the norms (for age and gender), the result is transferred to a sten value calculation sheet, drawing a personality profile. Results are obtained in the following sten ranges: 1 and 2 –very low; 3 and 4 –low; 5 and 6 –average; 7 and 8 –high; 9 and 10 –very high. The Polish adaptation of this test is characterised by good psychometric indicators [23, 24]. Additionally, each of the respondents specified their gender by marking on the test sheets whether they are male or female. No additional options were given.

### Statistical analysis

Elements of descriptive statistics were applied in the analysis. The results were presented as the mean values for groups with a standard deviation or as a percentage. P-values below 0.05 were considered statistically significant. The statistical analysis was performed using Statistica 13 PL and PS IMAGO PRO (SPSS).

To assess the relationship between personality factors and gender and their interaction with the variable dependent level of empathy, linear regression analysis with the interaction component was used.

In order to analyse the interaction with the dichotomous moderator, a variable gender status of the subjects was introduced. Independent variables were individual personality factors, gender and personality × gender interaction. Independent variables were centred to reduce the multicollinearity, while for the personality variables the result standardization method was chosen. The analysis was carried out in two stages, first for major personality factors such as Neuroticism, Extraversion, Openness, Agreeability and Conscientiousness. In the second part, individual components of personality dimensions were analysed.

## Results

The subjects were aged 21 to 28, with an average age of M = 23.24 with standard deviation = 0.84, of which 63% were female. Tables 1 and 2 contain the mean and standard deviation values for Big Five personality factors and empathy factors. Table 3 contains the analysis of the interaction with dichotomous moderator (statistical significance).

**Table 1:** Mean and SD for Big Five personality factors residualized scores.

Variable	Group					
	Female		Male		Full sample	
	M	SD	M	SD	M	SD
*Personality factors*						
Neuroticism	5,34	2,43	5,26	2,83	5,31	2,58
Extraversion	5,37	2,30	5,35	2,40	5,36	2,33
Openness to experience	6,02	2,20	6,91	2,06	6,36	2,18
Agreeableness	6,02	2,67	5,44	2,61	5,80	2,65
Conscientiousness	6,70	2,32	7,11	2,46	6,86	2,37
*Neuroticism*						
Anxiety	5,78	2,41	5,35	2,77	5,61	2,56
Hostility, anger	4,90	2,33	5,19	2,41	5,01	2,36
Depression	5,58	2,65	5,12	2,71	5,41	2,68
Self-consciousness	6,45	2,57	6,21	2,71	6,36	2,62
Impulsiveness	6,35	2,41	6,00	2,54	6,22	2,46
Vulnerability to stress	4,66	2,40	4,53	2,69	4,61	2,51
*Extraversion*						
Kindness	6,21	2,66	5,84	2,45	6,07	2,58
Gregariousness	5,47	2,23	5,37	2,16	5,43	2,20
Assertiveness	5,55	2,63	5,88	2,75	5,68	2,67
Activity	5,68	2,45	5,93	2,40	5,78	2,43
Excitement seeking	4,60	2,14	4,79	2,14	4,68	2,14
Positive emotion	6,24	2,49	6,09	3,08	6,18	2,72
*Openness to experience*						
Imagination	6,07	2,45	6,49	2,01	6,23	2,29
Aesthetics	4,95	2,56	5,88	2,54	5,30	2,58
Emotionality	5,82	2,32	6,16	2,96	5,95	2,58
Actions	5,70	2,26	5,96	2,46	5,80	2,33
Ideas	6,47	2,35	7,70	1,81	6,95	2,24
Values	7,68	2,38	7,05	2,64	7,44	2,50
*Agreeableness dimension*						
Trust in others	6,05	2,85	6,18	2,69	6,10	2,78
Straightforwardness	5,97	2,40	5,98	2,62	5,97	2,48
Altruism	5,93	2,72	5,70	2,75	5,84	2,72
Compliance	6,47	2,09	5,33	2,04	6,03	2,14
Modesty	5,86	2,48	4,74	2,51	5,43	2,55
Tendermindedness	5,71	2,82	5,05	2,79	5,46	2,82
*Conscientiousness*						
Competence	7,43	2,42	7,75	2,31	7,55	2,37
Organizing	6,14	2,49	6,86	2,60	6,42	2,55
Dutifulness	6,84	2,07	7,18	2,13	6,97	2,09
Achievement Striving	6,40	2,62	7,35	2,69	6,76	2,68
Self-Discipline	6,08	2,44	6,47	2,65	6,23	2,52
Deliberation	6,88	2,22	6,44	2,19	6,71	2,21

**Table 2:** Mean and SD for Empathic Sensitiveness Scale residualized scores.

Variable	Group					
	Female		Male		Full sample	
	M	SD	M	SD	M	SD
*Empathic Sensitiveness Scales*						
Empathic Concern	5,65	2,07	5,72	2,29	5,67	2,15
Personal Distress	4,77	2,14	4,60	2,17	4,71	2,15
Perspective-taking	6,72	1,98	6,93	1,88	6,80	1,94

**Table 3:** The relationship between the level of empathy and personality factors.

*Main personality factors*							
Factor		Empathic Concern		Personal Distress		Perspective-taking	
	Variables	Beta	p	Beta	p	Beta	p
*Neuroticism*	Neuroticism	.044	.937	.574	**.000**	-.159	.055
	Gender	.007	.602	-.048	.481	.061	.467
	Neuroticism × Gender	-.040	.629	.005	.941	-.071	.387
*Extraversion*	Extraversion	.436	**.000**	-.236	**.005**	.201	**.017**
	Gender	.008	.912	-.058	.477	.063	.782
	Extraversion × Gender	.129	.094	-.018	.826	.142	.087
*Openness to experience*	Openness to experience	.442	**.000**	-.081	.364	.349	**.000**
	Gender	-.087	.266	-.041	.627	-.014	.859
	Openness to experience × Gender	.104	.194	.020	.815	.154	.064
*Agreeableness*	Agreeableness	.506	**.000**	.252	**.003**	.416	**.000**
	Gender	.059	.410	-.029	.724	.107	.163
	Agreeableness × Gender	-.034	.644	.030	.723	.002	.976
*Conscientiousness*	Conscientiousness	.146	.084	-.261	**.002**	.169	.043
	Gender	-.008	.920	-.034	.627	.045	.581
	Conscientiousness × Gender	-.121	.149	-.035	.665	.155	.063
*Neuroticism dimension*							
*Anxiety*	Anxiety	.126	.130	.544	**.000**	-.071	.393
	Gender	.015	.855	-.012	.869	.055	.511
	Anxiety × Gender	-.067	.420	.017	.810	-.098	.241
*Hostility*, *anger*	Hostility, anger	.023	.784	.281	**.001**	-.328	**.000**
	Gender	.004	.963	-.074	.360	.082	.299
	Hostility, anger × Gender	.054	.527	.004	.959	-.057	.954
*Depression*	Depression	.047	.583	.507	**.000**	-.051	.546
	Gender	.011	.900	-.012	.873	.056	.503
	Depression × Gender	.025	.771	.128	.086	-.120	.158
*Self-consciousness*	Self-consciousness	.022	.792	.521	**.000**	-.011	.898
	Gender	.007	.936	-.032	.657	.061	.465
	Self-consciousness × Gender	-.036	.672	.130	.076	-.104	.219
*Impulsiveness*	Impulsiveness	-.036	.673	.307	**.000**	-.219	**.009**
	Gender	.001	.987	-.034	.627	.046	.575
	Impulsiveness × Gender	-.143	.090	.074	.359	-.082	.325
*Vulnerability to stress*	Vulnerability to stress	-.090	.282	.558	**.000**	-.192	**.021**
	Gender	.004	.965	-.042	.544	.057	.485
	Vulnerability to stress × Gender	-.020	.815	.009	.902	-.030	.717
*Extraversion dimension*							
*Kindness*	Kindness	.676	**.000**	-.024	.787	.439	**.000**
	Gender	.055	.388	-.058	.485	.095	.212
	Kindness × Gender	.157	**.021**	-.014	.872	.146	.068
*Gregariousness*	Gregariousness	.296	**.002**	-.066	.446	.140	.102
	Gender	.012	.877	-.058	.487	.066	.427
	Gregariousness × Gender	.026	.759	.067	.433	.000	.998
*Assertiveness*	Assertiveness	.157	.064	-.406	**.000**	.057	.503
	Gender	-.004	.961	-.031	.684	.059	.482
	Assertiveness × Gender	.051	.547	-.068	.382	.013	.879
*Activity*	Activity	.290	**.001**	-.286	**.001**	.107	.201
	Gender	-.010	.899	-.042	.604	.054	.504
	Activity × Gender	.157	.059	-.054	.515	.266	**.002**
*Excitement seeking*	Excitement seeking	.061	.474	-.003	.970	-.092	.281
	Gender	.003	.974	-.056	.499	.066	.427
	Excitement seeking × Gender	.083	.333	.008	.929	.029	.734
*Positive emotion*	Positive emotion	.384	**.000**	-.136	.102	.199	**.014**
	Gender	.017	.827	-.061	.456	.069	.393
	Positive emotion × Gender	.021	.788	-.136	.099	.169	**.037**
*Openness to experience dimension*							
*Imagination*	Imagination	.158	.082	.171	.060	.069	.451
	Gender	-.007	.928	-.073	.376	.055	.510
	Imagination × Gender	-.037	.682	.065	.472	.045	.621
*Aesthetics*	Aesthetics	.392	**.000**	.053	.544	.251	**.003**
	Gender	-.066	.402	-.068	.424	.015	.859
	Aesthetics × Gender	.078	.329	.050	.563	.083	.323
*Emotionality*	Emotionality	.506	**.000**	.033	.689	.180	**.030**
	Gender	-.026	.722	-.058	.484	.050	.541
	Emotionality × Gender	-.010	.889	-.015	.859	.051	.531
*Actions*	Actions	.132	**.001**	-.396	**.000**	.200	**.016**
	Gender	-.003	.972	-.035	.651	.049	.543
	Actions × Gender	.135	.105	-.011	.886	.170	**.039**
*Ideas*	Ideas	.180	.063	-.224	**.020**	.268	**.005**
	Gender	-.044	.614	.003	.974	-.016	.851
	Ideas × Gender	.028	.766	.010	.915	.102	.268
*Values*	Values	-.042	.617	.024	.776	.120	.156
	Gender	.001	.987	-.055	.516	.077	.358
	Values × Gender	.018	.829	-.038	.652	-.007	.929
*Agreeableness dimension*							
*Trust in others*	Trust in others	.366	**.000**	.006	.941	.371	**.000**
	Gender	-.002	.982	-.056	.498	.054	.488
	Ideas × Gender	.028	.726	-.044	.609	.088	.282
*Straightforwardness*	Straightforwardness	.247	**.003**	.275	**.001**	.128	**.000**
	Gender	.003	.946	-.057	.472	.067	.452
	Straightforwardness × Gender	-.126	.121	.126	.122	-.078	.348
*Altruism*	Altruism	.617	**.000**	.073	.390	.376	**.000**
	Gender	.032	.629	-.053	.522	.079	.310
	Altruism × Gender	.022	.740	-.016	.848	.096	.230
*Compliance*	Compliance	.239	**.007**	.157	.074	.419	**.000**
	Gender	.067	.431	-.009	.918	.166	.065
	Compliance × Gender	-.020	.816	.105	.218	-.057	.468
*Modesty*	Modesty	.275	**.001**	.352	**.000**	.272	**.001**
	Gender	.062	.449	.021	.798	.117	.154
	Modesty × Gender	-.054	.508	.032	.692	-.067	.412
*Tendermindedness*	Tendermindedness	.506	**.000**	.137	.110	.328	**.000**
	Gender	.063	.385	-.042	.615	.099	.214
	Tendermindedness × Gender	-.056	.450	-.038	.658	-.061	.447
*Conscientiousness dimension*							
*Competence*	Competence	.131	.132	-.393	**.000**	.147	.088
	Gender	.003	.967	-.029	.706	.052	.532
	Competence × Gender	.053	.537	-.063	.436	.044	.605
*Organizing*	Organizing	.182	.107	-.202	**.016**	.068	.418
	Gender	-.017	.833	-.025	.759	.047	.570
	Organizing × Gender	.150	.074	.101	.224	.184	**.029**
*Dutifulness*	Dutifulness	.174	**.040**	-.125	.142	.150	.077
	Gender	-.007	.930	-.047	.572	.049	.551
	Dutifulness × Gender	-.027	.751	-.014	.867	.060	.476
*Achievement Striving*	Achievement Striving	.085	.327	-.163	.058	-.008	.923
	Gender	-.012	.888	-.028	.739	.059	.482
	Achievement Striving × Gender	.081	.340	-.006	.948	.107	.209
*Self-Discipline*	Self-Discipline	.077	.356	-.370	**.000**	.096	.249
	Gender	.003	.973	-.028	.719	.052	.528
	Self-Discipline × Gender	.170	**.043**	.010	.896	.179	**.032**
*Deliberation*	Deliberation	.046	.591	.070	.415	.271	**.001**
	Gender	.013	.877	-.050	.551	.090	.267
	Deliberation × Gender	.102	.236	-.009	.918	.058	.488

The analysis showed that Extraversion, Openness and Agreeableness are associated with the level of Empathic Concern. Neuroticism, Extraversion, Agreeableness and Conscientiousness are associated with the level of Personal Distress. Extraversion, Openness, Agreeableness and Conscientiousness are associated with the level of Perspective-taking. The regression analysis with the interactive component showed that there is no relationship between gender and the level of empathy, and the interactions were insignificant.

In the second part, the relationship between the components of Neuroticism and the variable dependent level of empathy was analysed. The independent variables were the components of Neuroticism (Anxiety, Aggressive Hostility, Depression, Self-consciousness, Impulsiveness and Vulnerability to stress), gender, and the Neuroticism dimension x gender interaction. The analysis showed that Anxiety, Aggressive Hostility, Depression, Self-consciousness, Impulsiveness and Vulnerability to stress are associated with the level of Personal Distress. Aggressive Hostility, Impulsiveness and Vulnerability to stress are associated with the level of Perspective-taking. No significant result was found for the relationship between gender and level of empathy. None of the interaction analyses were significant.

Then, the relationship between the components of Extraversion and the level of empathy was analysed. The independent variables were the components of Extraversion (Kindness, Gregariousness, Assertiveness, Activity, Experience Seeking and Positive Emotions), gender, and the Extraversion dimension × gender interaction. The analysis showed that Kindness, (gender x kindness x the Empathic Concern) Gregariousness, Activity and Positive Emotions are associated with a level of Empathic Concern. Assertiveness and Activity are associated with a level of Personal Distress. Kindness and Positive Emotions are associated with the level of Perspective-taking. No significant result was found for the relationship between gender and level of empathy. However, the effect of interaction between Kindness and gender and Empathic Concern turned out to be significant. Interaction analysis showed that the relationship between Kindness and Empathic Concern is stronger in a group of men. Moreover, the interaction between Activity and gender and Perspective-taking and the interaction between Positive Emotions and gender and Perspective-taking was also important. The relationship between Activity and Positive Emotions and Perspective-taking varies by gender. The higher the level of Activity and Positive Emotions the higher the level of Perspective-taking in a group of men. No such relationship was observed in the group of women (see Table 1). All models were well matched to the data for the Kindness component F (3, 144) = 31.8; p <0.001, the Activity component F (3, 144) = 3.7; p <0.5 and the Positive Emotions component F (3, 144) = 3.6; p <0.5.

In the next part, the relationship between the components of Openness to experience and the dependent variable level of empathy was analyzed. The independent variables were the components of Openness to experience (Imagination, Aesthetics, Emotionality, Actions, Ideas and Values), gender, and the Openness to experience dimension × gender interaction. The analysis showed that Aesthetics, Emotionality and Actions are associated with the level of Empathic Concern. Action and Ideas are associated with a level of Personal Distress. Aesthetics, Emotionality, Actions and Ideas are related to the level of Perspective-taking. No significant result was found for the relationship between gender and level of empathy. The interaction between Action and gender and Perspective-taking was important. The relationship between Action and Perspective-taking varies by gender. The higher the level of Action, the higher the level of Perspective-taking in a group of men. No such relationship was observed in the group of women (see Table 1). The model was well matched to the data for Action component F (3, 144) = 3.2; p <0.5. In the next part, the relationship between the components of Agreeableness and the dependent variable level of empathy was analysed. The independent variables were the components of Agreeableness (Trust in others, Straightforwardness, Altruism, Compliance, Modesty, Tendermindedness), gender and the Agreeableness × gender interaction. The analysis showed that Trust in others, Straightforwardness, Altruism, Compliance, Modesty and Tender Mindedness are associated with the Empathic Concern. Straightforwardness and Modesty are associated with the level of Personal Distress. Trust in others, Straightforwardness, Altruism, Compliance, Modesty and Tender Mindedness are associated with the level of Perspective-taking. The regression analysis with the interactive component showed that there is no relationship between gender and the level of empathy, and the interactions were insignificant.

To conclude, the relationship between the components of Conscientiousness and the dependent variable level of empathy was analysed. Then, the relationship between the components of Conscientiousness and the dependent variable level of empathy was analysed. The independent variables were the components of Conscientiousness (Competence, Organizing, Dutifulness, Achievement Striving, Self-Discipline, Deliberation), gender and the Conscientiousness × gender interaction. The analysis showed that Dutifulness is associated with the Empathic Concern. Competence, Organizing, Self-Discipline are associated with the level of Personal Distress. Deliberation is associated with the level of Perspective-taking. The interaction between Organizing and gender and Perspective-taking was important. The relationship between Organizing and Perspective-taking varies by gender. The higher the level of Organizing, the higher the level of Perspective-taking in a group of men. No such relationship was observed in the group of women. The interaction between Self-Discipline and gender and Empathic Concern and Perspective-taking was also significant. The relationship between Self-Discipline and Empathic Concern and Perspective-taking varies by gender. The higher the level of Self-Discipline, the higher the level of Empathic Concern and Perspective-taking in a group of men. No such relationship was observed in the group of women (see Table 1). All models were well matched to the data for the Organizing component F (3, 144) = 3.8; p <0.05, the Self-Discipline F (3, 144) = 3.4; p <0.5 and the Self-Discipline component F (3, 144) = 3.2; p <0.5.

## Discussion

The main objective of the study was to analyse personality related conditions of empathetic sensitivity in students of medicine in relation to gender differences. It is worth mentioning, that in our previous research on the same group of students [20] no differences were observed in the sten levels of empathetic sensitivity in women and men. A number of empirical research applying tools based on Davis’s theory and the model of Big Five by Costa and McCrae have been carried out around the world [6, 8, 25, 26]. This study is original because the analysis of empathy determinants is further detailed to encompass components of basic personality factors in a homogeneous group of students in their fifth year of medicine studies.

### Empathetic concern

The first analysed dimension of empathy is Empathetic Concern (EC). EC has positive correlation with the level of Extraversion factor (E), and specifically with its components: Kindness (E1), Gregariousness (E2), Activity (E4), Positive emotions (E6). Guilera’s research team [8], has been testing interactions between personality factors and empathy in a group of Spanish students of medicine with results similar to ours. Extrovertive subjects are described as kind, gregarious, experiencing positive emotions and pleasant emotional states. They are active, focused on the external world rather than the inner world and seeking contact with others [26]. The ability to experience friendly feelings or warmness towards others plays a key role in situations when it is necessary to take empathetic care of another [27]. Nancy Eisenberg [28] links high empathy with mature prosocial moral reasoning, i.e. intellectual aspect of morality, related to caring for others. This stance, together with empathic care and compassion, is linked with the ability to concentrate on another person, with temporary inhibition of one’s own needs [22].

There is evidence showing that gender related differences in the levels of empathy are rooted in ontological and phylogenetical biological factors, as well as environmental influences and socialisation process [15, 29, 30]. Some authors hypothesise that they are related to both, external factors (such as expectations embedded in social roles) and internal factors (such as biological characteristics and aspects of neurological functioning).

A higher level of EC is related to a higher level of Openness to experience (O) and its components Aesthetics (O2), Emotionality (O3), Action (O4) [comp. Song and Shi, 2017]. The following interpretation of this result is that readiness to introduce changes in their environment, makes subjects more sensitive to others and their emotional states and enhances their efforts to reduce their pain [31].

EC and Agreeableness (A) and all its components Trust in others (U1), Straightforwardness (U2), Altruism (U3), Compliance (U4), Modesty (U5), Tender Mindedness (U6) are positively correlated. It means that subjects who often take care of others may have the need to resign from their own desires in order to fulfil the needs of others. High level of Compliance makes them more sensitive to expectations, desires and emotions of others than their own. Results of this study are convergent with results in a group of Chinese [6] and Spanish [8] students of medicine. Portugues research [32, 33] show a high relationship between (O) and (A) factors and general level of empathy and proposes that (O) and (A) are key personality factors of medical students in the clinical professional context.

It has been noted that subjects with high Dutifulness (S3) score higher results in Empathetic Concern EC.

EC is positively related to Self—discipline (S5) only in male subjects. It seems that all components of the Conscientiousness (S) factor, refer to cognitive functioning. The prevalence of positive correlation in male vs female group may be explained by the fact that areas of the brain responsible for cognitive control of empathy are more strongly activated in males rather than females [29].

### Personal distress

The other affective indicator of empathy is Personal Distress (PD). No correlation between PD and gender has been discovered, which means that there is no difference between male and female students of medicine in this area. However, PD has been related to four other main personality factors: Neuroticism (N), Extraversion (E), Agreeableness (A), Conscientiousness (C) and their components. The higher results in N and all its components, the more unpleasant feelings the subjects experienced when faced with suffering of other people. Similar results have been observed in another study of medical students [6, 8] or volunteers taking care of seriously ill patients [9]. The relationship between neuroticism and susceptibility to experiencing unpleasant feelings seems to be intuitive. Both constructs refer to similar areas of human emotional functioning. According to the definition proposed by McCrae and Costa [34], neuroticism is a personality related disposition to excessive experiencing of negative feelings such as anxiety, distress and danger in relationships as well as impulsiveness and difficulties in constructive coping with stress. Neuroticism is accompanied by a difficulty to efficiently regulate emotions. This disposition, together with the ineffective process of affective self–regulation comprise the definition of “Personal Distress” [5, 35]. It is highly probable–and confirmed by our own research and above mentioned results of other authors, [6, 8]–that an individual who, for personality related reasons, is sensitive to remaining in an emotional state characterized by emotional tension, will be highly susceptible to similar emotional reactions when faced with situations perceived as difficult.

Personal distress and Extraversion (E) are negatively correlated [6, 8]. Extraverted individuals are more susceptible to experiencing positive emotional states [26], which may be related to general minimisation of negative emotions in their emotional life. Personal distress is related to temporarily focusing on one’s own emotional states, desire to reduce unpleasant tension and taking care of oneself [22, 36]. Compared to introverted individuals, subjects with a high level of extraversion are probably less susceptible to concentrate on their own Self.

A positive correlation between levels of Agreeableness (A), with its components, and PD has been observed. Highly agreeable individuals are exposed to numerous frustrations resulting from the tendency to resign from meeting their own needs in relationships and focusing on expectations of the other person. Therefore, it may be hypothesised that highly agreeable individuals will be more vulnerable to experiencing unpleasant feelings when they are in contact with people who are suffering. In the research conducted among the group of Spanish [8] and Chinese medical students [6] a contrary dependence has been observed–strong negative correlation between factor A and the PD indicator. It raises the question about the influence of a certain culture on different research results. Previous studies indicate that culture plays its role when it comes to the A factor as Chinese and other East Asian students score lower in agreeableness compared with other samples [6].

Costa [32], who examined students from three medical schools in Portugal, argues that there is no correlation between Conscientiousness (C) and empathy, explaining that both constructs are independent, although Conscientiousness seems to be the key aspect of being a medical doctor. Differently to Costa, results of this research, indicate that there is a correlation between these factors. Conscientiousness (C) and its components Competence (C1), Organizing (C2), Self—discipline (C5) have negative correlation with PD. It may indicate that competent, efficient and organised individuals experience less Personal Distress PD and can act despite frustrations. This result is coherent with results of earlier analyses conducted among students of medical university in Northeast China and among students of the Faculty of Medicine in Spain [6, 8].

### Perspective taking

Perspective Taking (PT) factor has positive correlation with Extraversion (E) and its components—Kindness (E1), Positive emotions (E6) that describe positive relations with people and the tendency to experience positive emotions [6, 8]. Extraversion supports directing attention and emotional engagement beyond the inner experiences of an individual, strengthening aspects of empathy, that are related to temporary resignation from focusing on inner states. Positive correlation with EC and negative correlation with PD seem to give support to that hypothesis. Two components of E—Activity (E4) and Positive emotions (E6), are correlated with male gender.

The PT factor is correlated with four components of the Openness to experience (O) factor—Aesthetics (O2), Emotionality (O3), Action (O4), Ideas and Values (O5). Openness to emotional experiences of others, together with awareness of own emotional states, flexibility in thinking and action taking are strongly correlated with understanding of inner experiences such as intentions, beliefs, emotions and needs of other people [31, 36]. A better understanding of others favours action taking. It has been confirmed in male subjects group in this study by correlation between PT and O4. Previous studies [6, 8] confirm these results as well.

Our research has shown that PT has positive correlation with Agreeableness (A) and all its components [6, 8]. Therefore we can presume that a specific attitude together with cognitive openness enhance understanding of the other’s perspective, coming closer to them, some level of compliance and withdrawal of their own perspective. Melchers [25] indicates that A is the most important personality factor that explains PT and EC dimensions. The quoted research was carried out in a group of medical students from China, Germany, Spain and the USA. Song and Shi [6] stress that Agreeableness and empathy are related on the level of neurophysiological structures by the activity of mirror neurons [37, 38]. Their activity is related to processes of understanding emotions, intentions, states and further interpretation of other’s activities. This is an interesting hypothesis, although in this study it is not central, so it is mentioned only.

PT is negatively correlated with three components of Neuroticism: Aggressive hostility (N2), Impulsiveness (N5), Vulnerability to stress (N6). All these components are related to concentration on one’s own internal emotional states. The higher the level of experiencing anger and distress, the more difficult it is to concentrate on the other’s perspective. The higher the level of impulsivity and accompanying difficulty in emotional control, the lower the reflexivity and the ability to focus attention on another person and the lower the engagement of cognitive function of empathy. A high level of oversensitivity is responsible for excessive concentration on one’s own emotions and limits the ability to concentrate on the surrounding environment. This interpretation is further supported by the fact that PT is positively related to another component of Conscientiousness–Deliberation (C6). Perspective taking, as a cognitive component of empathy, is related to cognitive analysis of the situation.

The following gender related differences were observed in the group of male subjects. The higher the level of Organizing (C2), the higher the factor Perspective taking was. Christov-Moore [29] have shown that the areas of the brain engaged in cognitive control of empathy are more strongly activated in men than in women. Correlation in male subjects’ group and its lack in female subjects’ group is coherent with these results.

## Conclusions

The most important conclusions from our study are the following:

- Level of empathy is related to personality dimensions of medicine students.
- No interactions were discovered between five main factors of personality, three indicators of empathy and gender.
- Detailed analysis of personality factors’ components shows gender related differences.

In further education of medical doctors it is worth remembering, that the willingness to experience and express empathy may change the culture of medicine–from indifferent, fragmentary interest, limited to the object of treatment–to a broader subject oriented, encompassing the patient’s perspective. Empathy in modern clinical care may be understood as a process of emotional resonance and interest in the patient’s meaning of the clinical situation. Medical doctors need courage to enter the interpersonal world and train their empathetic skills. Empathy changes the way medical doctors experience their job–from limited to medical activities–to a vocation that gives meaning to their lives [39]. It is therefore not surprising that the issue of medical doctors’ personality and its relations to empathy are broadly discussed in the professional literature. Effectiveness of doctors’ interventions–health and life of their patients, may depend on their personality expressed also through empathy. These observations are coherent with narrations given by subjects of the study. Interviews with students of medicine who participated in this study clearly show the meaning and value of empathy in their further professional development and everyday clinical work.

Thus our research is of practical importance in the process of educating future doctors. Understanding the importance of personality factors in relationship with medical empathy among medical students may contribute to the enrichment of educational methods and training programs aimed at enhancing students’ empathy in medical education. The problem is especially important because, as some studies show, the level of empathy shows a worrying decline in the course of medical education [40]. It is worth to add that personality and empathy-targeted interventions have gained increasing popularity in health promotion research and practice.

The presentation of our research results in workshops for medical students will contribute to the increase of students’ ’awareness of the relationship between their unique personality traits and individual empathy dimensions and subsequent translation of them into the doctor-patient communication, with a better understanding of patients’ experiences, concerns, and perspectives.

Moreover, this knowledge may contribute to a more conscious choice of the further education path and type of specialization, including those more technology oriented specialties or people-oriented specialties.

## Limitations

There are a few limitations in our study. Empathy has been measured by means of self-descriptive methods and not evaluated in natural clinical situations. A self-descriptive tool has been applied in personality testing, and therefore it provides a subjective perspective of subjects. The fact that the group of subjects comprises a relatively homogenous group of fifth year’s students of medicine may be considered as a limitation because it precludes extrapolation of results on all students of medicine. The division of our group to only two categories: male and female, with no additional options, might have limited the potential results.
